# Exploring the relationship between migrants’ purchasing of commercial medical insurance and urbanisation in China

**DOI:** 10.1186/s12913-018-3503-1

**Published:** 2018-09-03

**Authors:** Jinhua Liu, Hongsheng Chen, Yang Chen, Zhigang Li

**Affiliations:** 1 School of Architecture, Southeast University, No.2, Sipailou Road, Xuanwu District, Nanjing, 210096 China; 2 School of Urban Design, Wuhan University, Ba Yi Road No.299, Wuhan, 430072 China

**Keywords:** Commercial health insurance, Migrants, Urbanisation, Neighbourhood, China

## Abstract

**Background:**

Over the past 40 years, the Chinese government has diligently promoted the market-oriented reform of the health insurance system. However, as it is affected by the urban-rural dual structure, most rural-to-urban migrants are excluded from local public health services and medical insurance schemes in the cities in which they live. Buying local commercial medical insurance (CMI) is an important way for migrants to obtain local medical services. Therefore, this study’s purposes were to explore the city factors that affect migrants’ purchase of CMI and to investigate the relationship between urbanisation and migrants’ purchase of CMI.

**Methods:**

This study used the 2013 National Floating Population (Migrants) Dynamic Monitoring Survey data, which covered 31 provinces, municipalities, and autonomous regions in China. The respondents were migrants (15–59 years; *n* = 164,752) who lived in the inflow areas for more than a month without obtaining a local household registration record. We used city factors, neighbourhood factors, and individual factors that affect residents’ purchase of CMI to construct a theoretical framework and examined the effect of urbanisation on migrants’ choice of CMI using multilevel logistic regression.

**Results:**

The proportion of migrants who had local or hometown CMI was 5.70%; most migrants had no CMI (94.30%). Of these, 4.42% had CMI in the city in which they live (i.e. local CMI) and 1.64% had CMI in their hometown (i.e. hometown CMI). Migrants living in first-tier and third-tier cities were less likely to have CMI than those in second-tier cities (odds ratio [OR] = 0.454, 95% confidence interval [CI]: 0.395–0.521; OR = 0.588, 95% CI: 0.464–0.746). Furthermore, the regression results revealed a U-shaped relationship between the odds of migrants having CMI and the urbanisation rate of the prefecture-level cities. The findings also indicated that with higher socioeconomic status, there was a greater probability of purchasing CMI.

**Conclusions:**

The improvement of urbanisation has a positive effect on migrants’ purchase of CMI. However, China’s medical insurance market is still underdeveloped. It is necessary for the Chinese government to increase migrants’ participation rate in CMI to cover soaring medical expenses.

## Background

Since the Chinese government implemented its policy of reform and opening up 40 years ago, a large number of rural residents have moved to urban areas and China’s urbanisation rate has risen rapidly. The number of rural-to-urban migrants in China reached 245 million and China’s urbanisation rate reached 57.4% in 2016 [1]. Meanwhile, over the past 40 years, the Chinese government has worked hard to promote the market-oriented reform of the economic and social security system to increase the efficiency of economic development and reduce the government’s burden on public finance [2, 3]. The medical security system is one of the Chinese government’s important targets for implementing market-oriented reform [4, 5].

There have been quite a few studies of the reform of China’s health insurance system. For example, Liu [6] found that after the health insurance system reforms in urban China, the new insurance system has expanded coverage to private sector employees and it provides more stable financing with its risk pool at the city level. However, some researchers have pointed out the shortcomings of China’s health insurance reform [4, 7, 8]. Hsiao [9] proposed that the market-oriented reform of the Chinese macro-health policy has resulted in uncoordinated financing, pricing, and organisational policies that exacerbate inequity and inefficiency in the health care. Especially, the unequal health insurance policy for urban residents and rural residents has been criticised by many scholars [10]. Using the 2006 China Agricultural Census, Chen and Jin [11] found that the introduction of the New Cooperative Medical System (NCMS) in rural China does not affect child mortality or maternal mortality. Lei and Lin [12] reported that participating in the NCMS does not lead to decreased out-of-pocket expenditure and it increases the utilisation of the formal medical service and improves the health status in rural China.

Commercial medical insurance appeared in China in the 1980s. However, after more than 30 years of development, the rate of purchase of commercial medical insurance (CMI) among Chinese residents remains very low and most Chinese rely on public medical insurance [13]. Compared with public medical insurance, CMI is more targeted and can provide residents with more diversified medical insurance options, such as critical illness insurance. Recently, China’s commercial insurance market has entered a period of rapid development. From 2010 to 2015, the market value of China’s CMI grew at an average annual rate of 36% and the market size of China’s CMI reached 36.7 billion US dollars in 2015 [14]. However, given the high price of CMI, most low- and middle-income residents are still less likely to purchase CMI, and CMI has not been widely accepted in China [15, 16]. The low purchase rate of CMI has exacerbated China’s health inequities. In particular, most rural-to-urban migrants are excluded from the local public health services and medical insurance schemes in the cities in which they live [17, 18]. Poor working and living conditions and inattention to health make migrants vulnerable to ill health [19, 20]. For instance, the research of Hong et al. [21] found that migrants’ lack of insurance coverage, the high cost of health care, and exacting work schedules have resulted in the use of unsupervised self-treatment and substandard health care. Peng et al. [22] proposed that the current health service system discourages migrant workers from seeking appropriate, high quality care. With the difficulties of changing the existing basic social medical insurance system, CMI has become important for enhancing the health of migrants [23]. However, there has been little research into whether migrants will buy CMI in China [15].

In developed countries, CMI plays an important role in the medical security system [24, 25]. As medical costs are high, residents rely on medical insurance to cover these costs. In addition, the health insurance coverage rate is relatively high. Residents’ use of medical services is significantly related to their insurance status [26], and residents who purchase CMI can obtain better medical services. However, many more immigrants and ethnic minorities are still uninsured compared to native citizens. For example, in America, most low-income residents receive Medicaid-covered services provided by the government [27]. However, fewer noncitizen immigrants have Medicaid or job-based insurance than native citizens [28], and immigrants have lower rates of both private insurance coverage and public insurance coverage than native citizens [29]. Furthermore, although the Medicaid programme has improved access for low-income people, Medicaid enrolees are less likely than privately insured patients to have a personal physician [30]. Some scholars [31, 32] have argued that the administrative criteria (e.g. federal legislation) for public programmes contribute to immigrants’ lower rates of insurance. Due to the high price of CMI, both China and the United States may be faced with the possibility that few immigrants or low-income groups will purchase it.

Moreover, there is a close relationship between cities’ urbanisation levels and residents’ health [33–35]. For example, using data from eight waves of the China Health and Nutrition Survey from 1991 to 2011, Miao and Wu [36] found that living in more urbanised areas increases the risk of acquiring chronic diseases. In addition, Liu et al. [37] reported that urbanisation leads to a significant and equitable increase in insurance coverage in China. However, few studies have examined the relationship between urbanisation and migrants’ use of health insurance. Therefore, this study aimed to answer the following two questions: which migrants buy CMI and what is the relationship between urbanisation and migrants buying CMI?

The remainder of the paper is organised as follows. In the following sections, we introduce the data and methods used in this study. In the next section, we describe the analysis of the survey data and the regression results of this study. Section “Discussion” contains a discussion of the results and Section “Conclusions” is the conclusion.

## Methods

### Study design and theoretical framework

This study focuses on the correlation between urbanization rate of the prefecture-level city and migrants’ purchase of commercial insurance in China. This study examines two related relationships. The first is the correlation between urbanization rate and migrants’ purchase of commercial insurance, and the second is whether migrants living in different types of cities have differences in the probability of purchasing commercial insurance. Therefore, we use large scale national questionnaire survey data and multilevel regression analysis to explore the influence of city factors on individual health behaviors.

**Fig. 1:**
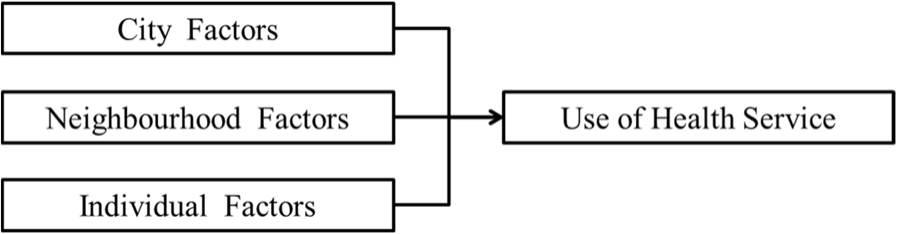
The three types of factors related to the use of health services

We modified Andersen’s behavioural model of health service utilisation to construct the theoretical framework of this study (see Fig. 1) [26, 38]. Previous studies have mainly focused on the impact of individual factors on the use of health services and have seldom considered the impact of external factors on the use of health services, such as city factors and neighbourhood factors [39, 40]. As such, we proposed that there are three types of factors that affect residents’ purchase of CMI: city factors, neighbourhood factors, and individual factors.

The city factors mainly concern the development level of the city, as there are huge differences in China in the quality of health services in cities of differing development levels. In this study, city factors included the variables of urbanisation rate and city type. The neighbourhood factors mainly concern the type of neighbourhood. We focused on the differences in the residents’ access to health services in different neighbourhoods as, in China, there is residential segregation based on neighbourhoods [41] and there are significant differences in the health level of residents in different types of neighbourhood [39, 42]. In this study, we examined the differences in the likelihood of migrants in urban neighbourhoods and rural neighbourhoods purchasing CMI. In addition, as previously established, residents’ socioeconomic status is closely related to their health status and health behaviours [43, 44]. Therefore, this study focused on the impact of city factors and neighbourhood factors on residents’ purchase of CMI. Consistent with existing research [34], this study used the individual factors as control variables to construct a regression analysis model.

### Data

This study used the 2013 National Floating Population (Migrants) Dynamic Monitoring Survey data to analyse the relationship between urbanisation and migrants’ purchase of CMI in China. This survey covered 31 provinces, municipalities, and autonomous regions in the whole country and was conducted by China’s National Health and Family Planning Commission in 2013. The respondents were migrants aged 15–59 years who had lived in the inflow areas for more than a month without obtaining a local hukou (i.e. a household registration, which is used to distinguish between locals and migrants in China [45]). The survey team selected respondents using a multistage, clustered, stratified, probability proportional-to-size sampling technique. After missing values were removed, a total of 164,752 valid sample members were used for our analysis. In addition, the urbanisation rate data were gathered from the China City Statistical Yearbook.

### Statistical analysis

We examined the effect of urbanisation on the migrants’ choice of CMI using multilevel logistic regression analysis (two levels: city level and individual level). We also used the chi-squared test and *t*-test to determine whether there is a significant difference between the migrants without local CMI and the migrants with local CMI in all independent variables and control variables. The dependent variable was a binary variable: a migrant who had CMI was coded as 1 and a migrant who did not have CMI was coded as 0. The independent variables included the urbanisation rate of the prefecture-level city, the type of prefecture-level city in which the migrants were living, and the type of neighbourhood in which the migrants were living.

The urbanisation rate of the prefecture-level city is the proportion of residents living in an urban area in the total population of the city. Generally speaking, cities with high proportion of residents living in an urban area in the total population have high level of urbanisation in China. In addition, given that the relationship between migrants having CMI and the level of urbanisation may be U-shaped rather than linear, we considered the square of the urbanisation rate in the regression models. In terms of the type of neighbourhood, although both the urban and rural neighbourhoods are in the same prefecture-level city, there is a large difference between their living environments and health services. The quality of the living environment and health services in rural neighbourhoods is far worse than in urban neighbourhoods. We divided the type of city into first-tier cities, second-tier cities, and third-tier cities based on the level of economic development [46]. The first-tier cities included Beijing, Shanghai, Guangzhou, and Shenzhen, which are China’s most prosperous cities. The second-tier cities included 45 cities that are mainly provincial capital cities and economically developed cities. The other cities were classified as third-tier cities. The living cost and public health services vary greatly across the different types of city. The control variables included the duration of residence in the city, marital status, gender, age, educational attainment, personal monthly income, occupation, average daily working hours, and hukou status. We run six regression models in this study. Model 1 and model 2 are the regression results of the impact of urbanisation on migrants having local CMI. Model 3 and model 4 are the regression results of the impact of urbanisation on migrants having hometown CMI. Model 5 and model 6 are the regression results of the impact of urbanisation on migrants having CMI (local CMI or hometown CMI). Stata 14.0 (Stata Corp., College Station, TX, USA) was used to perform the data analysis. The results were considered significant at the 5% level (*p* < 0.05).

## Results

### Descriptive statistics

Table 1 displays the proportion of migrants with CMI in the city in which they live and those with CMI in their hometown. The proportion of migrants with CMI (in both the city they are living in or in their hometown) was 5.70%; most migrants have no CMI in China (94.30%). Of these, the proportion of migrants with CMI in the city in which they live and the proportion with CMI in their hometown was 4.42% and 1.64%, respectively, while the proportion with CMI in both the city in which they live and their hometown was 0.37%.

Table 2 presents the descriptive statistics for the independent variables. Table 3 summarizes the results of cross-tabulations conducted of migrants with/without local commercial medical insurance. The results indicate that the greater the urbanisation rate of the prefecture-level cities, the higher the likelihood of migrants in the cities buying local CMI (*t*-test value = − 26.392, *p* < 0.001). The percentage of migrants with local CMI living in urban neighbourhoods (76.40%) was much larger than the proportion of those living in rural neighbourhoods (23.60%). Interestingly, the percentage of migrants with local CMI in the second-tier cities (46.17%) was much larger than that in the first-tier cities (19.14%) and third-tier cities (34.69%). The migrants with local CMI have stayed in the city longer than those without local CMI (5.13 years versus 4.52 years, *t*-test value = − 16.678, *p* < 0.001). The percentage of married migrants with local CMI was larger than that of single and divorced migrants (77.85% versus 22.15%, *p* < 0.001). The higher the education level of the migrants, the more likely they were to purchase local CMI. The proportion of migrants with local CMI who graduated from primary school or below, junior high school, senior high school, and college or above are 6.72%, 44.31%, 27.54%, and 21.43% (*p* < 0.001), respectively. Migrants with local CMI had a higher personal monthly income than those without local CMI (4,217.51 yuan versus 3,224.26 yuan, *t*-test value = − 36.323, *p* < 0.001). The better the occupation, the more likely the migrants were to buy local CMI (*p* < 0.001). The average daily working hours of migrants with local CMI were shorter than of those without local CMI (9.14 h versus 9.44 h, *t*-test value = 13.291, *p* < 0.001). In addition, the percentage of migrants with local CMI holding a non-agricultural hukou was larger than those with an agricultural hukou (73.61% versus 26.39%, *p* < 0.001).

### The relationship between migrants with CMI and urbanisation in China

Model 1 of the multilevel logistic regression was used to examine the impact of urbanisation on respondents having CMI in the city in which they are living (having local CMI). The odds of respondents having local CMI increased with the urbanisation rate of the prefecture-level cities (odds ratio [OR] = 1.016, 95% confidence interval [CI]: 1.013–1.020; Table 4). Furthermore, respondents living in the first-tier cities and third-tier cities were 0.454 times less likely (OR = 0.454, 95% CI: 0.395–0.521) and 0.588 times less likely (OR = 0.588, 95% CI: 0.464–0.746), respectively, to have local CMI than those living in the second-tier cities. Respondents living in rural neighbourhoods were 0.842 times less likely to have local CMI than those living in urban neighbourhoods (OR = 0.842, 95% CI: 0.791–0.897).

In terms of the control variables, the odds of respondents having local CMI increased with the duration of residence in the city (OR = 1.039, 95% CI: 1.034–1.044). Compared with single and male respondents, married and female respondents were 10.9% and 11.9% more likely to have local CMI (married respondents, OR = 1.109, 95% CI: 1.030–1.195; female respondents, OR = 1.119, 95% CI: 1.062–1.179), respectively. Respondents with higher educational attainment were more likely to have local CMI than those who had a primary school education or below (junior high school, OR = 1.458, 95% CI: 1.319–1.611; senior high school, OR = 2.074, 95% CI: 1.861–2.312; college and above, OR = 2.625, 95% CI: 2.318–2.972). Compared with respondents who were service staff, except for respondents with informal employment, the respondents with other occupations were more likely to have local CMI (manager, OR = 1.571, 95% CI: 1.254–1.96; technician, OR = 1.146, 95% CI: 1.054–1.246; production personnel, OR = 1.121, 95% CI: 1.054–1.191). In addition, respondents with informal employment were 0.682 times less likely to have local CMI than those who were service staff (OR = 0.682, 95% CI: 0.574–0.811). The odds of respondents having CMI decreased with an increase in their average daily working hours (OR = 0.940, 95% CI: 0.926–0.954). Compared with respondents with an agricultural hukou, respondents with a non-agricultural hukou were 0.245 times more likely to have local CMI (OR = 1.245, 95% CI: 1.165–1.331).

In terms of regional differences, migrants living in the eastern region of China were more likely to have local CMI than those in other regions of China (northeast region, OR = 0.537, 95% CI: 0.372–0.775; western region, OR = 0.711,95% CI: 0.555–0.912; central region, OR = 0.662, 95% CI: 0.493–0.889). Furthermore, when we added the quadratic forms of the urbanisation rate in Model 2, we found that the variable of the quadratic forms of the urbanisation rate was not statistically significant. The rest of the results of Model 2 were similar to those of Model 1.

Model 3 of the multilevel logistic regression was used to investigate the impact of urbanisation on respondents having CMI in their hometown. The variables of the urbanisation rate of the prefecture-level cities, the type of prefecture-level city, and the type of neighbourhood all have no significant effect on migrants having hometown CMI. In terms of the control variables, the odds of respondents having hometown CMI increased with their age (OR = 1.015, 95% CI: 1.009–1.021). Respondents with higher educational attainment were more likely to have hometown CMI than those who had primary school education or below (junior high school, OR = 1.414, 95% CI: 1.219–1.639; senior high school, OR = 1.829, 95% CI: 1.554–2.152; college and above, OR = 1.899, 95% CI: 1.564–2.306). Compared with respondents who were service staff, the respondents with other occupations (except managers) were less likely to have hometown CMI (technicians, OR = 0.820, 95% CI: 0.708–0.950; production personnel, OR = 0.759, 95% CI: 0.685–0.841; informal employment, OR = 0.761, 95% CI: 0.594–0.975). In addition, compared with respondents with an agricultural hukou, respondents with a non-agricultural hukou were 0.700 times more likely to have hometown CMI (OR = 1.700, 95% CI: 1.533–1.885). Migrants living in the northeast region of China were 0.579 times less likely to have hometown CMI than those living in the eastern region of China (OR = 0.579, 95% CI: 0.396–0.848). We also added the variable of the square of the urbanisation rate of the prefecture-level cities into Model 4. However, neither the urbanisation rate nor the square of the urbanisation rate was significant.

Model 5 and Model 6 of the multilevel logistic regression examined the impact of urbanisation on respondents having CMI (having local CMI or hometown CMI). In Model 5, we found that the odds of respondents having CMI increased with the urbanisation rate (OR = 1.009, 95% CI: 1.006–1.012). We added the quadratic forms of the urbanisation rate in Model 6 and found that both the quadratic forms and linear forms were statistically significant (urbanisation rate: *p* < 0.01; square of the urbanisation rate: *p* < 0.01). These results demonstrate a significant U-shaped relationship between the odds of respondents having CMI and the urbanisation rate of the prefecture-level cities.

## Discussion

Due to the urban-rural gap in China’s society and economy [47, 48], there is a huge difference between the medical insurance system for urban residents and rural residents (including migrants) [49]. In particular, most migrants do not have adequate access to health care services and are more vulnerable to health risks (e.g. environmental pollution and dangerous working conditions) [50, 51]. As an important supplement to China’s public health insurance, an increasing amount of insurance companies are providing CMI to the Chinese. However, this study demonstrates that most migrants do not buy CMI. The proportion of migrants with local/hometown CMI was 5.70%, and most migrants do not have CMI in China (94.30%). Unlike in Western countries, in which most residents rely on CMI (i.e. private medical insurance) to pay for health care [52], the popularity of CMI is still very low in China. On the one hand, this is related to the underdevelopment of the marketisation of China’s medical insurance market, and the quality and reputation of health insurance providers is a particular concern [53]. Therefore, many rich mainland Chinese people buy CMI and other medical services in Hong Kong (e.g. as medical tourists) instead of in mainland China [54]. On the other hand, the purchase of CMI is also affected by the residents’ socioeconomic status; most migrants of low socioeconomic status are less likely to buy CMI.

There was also a huge difference in the probability of migrants having CMI in different cities. From the regression results, we found that, in cities with high urbanisation rates, migrants were more likely to purchase CMI (local CMI or hometown CMI). This finding demonstrates that the likelihood of migrants buying CMI is affected by the development level of the city in which they live. In China, cities with high urbanisation rates are more market-oriented than those with low urbanisation rates and migrants working in cities with high urbanisation rates can earn more money than those working in cities with low urbanisation rates, which is related to the migrants’ purchase of CMI. However, we also found that the relationship between the urbanisation rate and the purchase of CMI by migrants is U-shaped. That is, migrants in the cities with the middle urbanisation rate were the least likely to buy CMI. This phenomenon may occur because migrants in cities with high urbanisation rates can afford CMI while migrants in the relatively economically disadvantaged cities may obtain more health benefits from the government.

This study provides several policy recommendations for China’s health care system. First, at present, the migrants who purchase CMI account for a very low proportion of the total population, and the coverage rate of CMI is relatively low. CMI is an important component of China’s medical insurance system, and it is necessary to increase the coverage rate of migrants’ CMI and for CMI to play a greater role in improving residents’ health. On the one hand, the price of CMI should be lowered so that migrants can afford it. On the other hand, China’s current CMI reimbursement procedures are complex and should be simplified. Second, there are great differences in the probability of migrants purchasing CMI in different cities. Migrants are less likely to buy CMI in less urbanised cities. Improving the availability and affordability of CMI in economically disadvantaged areas is an important way to narrow the gap in the health equity in China. Third, it is necessary to raise migrants’ awareness of health protection. Faced with the difficulty and expense of medical services in China, it is necessary to increase the participation rate of migrants in CMI to cover their medical expenses. In addition, it is urgent to protect the rights of those who have purchased CMI to increase migrants’ purchase intention.

There are also three research limitations in this study. First, residents who purchased CMI could have been affected by many different factors. For example, the substitutability of other medical insurance schemes (e.g. the basic social medical insurance) could reduce the possibility of residents purchasing CMI. Second, this study focused on the relationship between urbanisation and the purchase of CMI but did not explore the causal relationship underlying the purchase of CMI. Third, due to the data limitations, we did not examine the differences between locals and migrants who purchase CMI. However, overall, the socioeconomic status of locals is higher than that of migrants [55]. As such, the proportion of the locals who have CMI may be larger than that of migrants.

## Conclusions

This study contributes to the understanding of the impact of urbanisation on migrants’ health insurance choice. Using multilevel logistic regression and the data of the 2013 National Floating Population Dynamic Monitoring Survey, we found that there is a significant linear relationship between the odds of migrants having local CMI and the urbanisation rate of the prefecture-level cities in which they are living. In addition, there is a U-shaped relationship between the odds of migrants having CMI (local CMI or hometown CMI) and the urbanisation rate of the prefecture-level cities. Migrants living in the second-tier cities in China were more likely to have CMI than those living in the first-tier and third-tier cities. We also demonstrated that the higher the socioeconomic status of the migrants, the more likely they were to purchase it. This result indicates that the improvement of urbanisation has a positive effect on people’s purchasing of CMI.

## Abbreviations

CI: Confidence interval
CMI: Commercial medical insurance
NCMS: New Cooperative Medical System
OR: Odds ratio
